#  Association of Cytokine Gene Alleles with the Inflammation of Human Periodontal Tissue 

**Published:** 2011

**Authors:** A.V. Safonova, A.N. Petrin, S.D. Arutyunov, V.N. Tsarev, L.A. Akulenko, A.O. Zorina, D.V. Rebrikov, A.V. Rubanovich, S.A. Borinskaya, N.K. Yankovsky

**Affiliations:** Moscow State University of Medicine and Dentistry; Central Research Institute of Dentistry and Oral Surgery, Federal Agency of Medical Technologies; DNA-Technology JSC; Vavliov Institute of General Genetics, Russian Academy of Sciences

**Keywords:** gingivitis, periodontitis, cytokine genes, genetic polymorphism

## Abstract

Gingivitis and periodontitis are chronic inflammatory diseases of the periodontal tissue in humans caused by both environmental and genetic factors. The human cytokine genes that regulate the immune response may play an important role in the development of these chronic inflammatory diseases. The aim of this study is to analyze the allele status of eight human cytokine genes and to associate it with the inflammation of periodontal tissue in humans. A total of 296 unrelated males of Russian origin were studied. A significant association of the*IL1B*and*IL6 *minor alleles and gingivitis was found. In addition, we found a significant association of the OHI-S index with the*IL18*gene alleles. The influence of genetic factors on gingivitis may contribute to the understanding of the mechanisms of interaction between genetic and environmental factors in periodontal conditions, and to the identification of risk groups for effective prevention and treatment.

##  INTRODUCTION 

Gingivitis (inflammation of the gum tissue) and periodontitis (inflammation of tissues surrounding and supporting the teeth) are widespread oral diseases. Gingivitis can appear both in tandem with periodontitis and independently. Signs of periodontal tissue involvement might appear even in children 6—7 years old [1]; these indicators are found in more than 50% of 15-year-old adolescents, and the prevalence of periodontal diseases among adults in Moscow and other large cities reaches 98%. Moreover, symptoms of gingivitis are found in more than 80% of people [2].

 The inflammation and the destruction of tissues in periodontal diseases occur due to a disequilibrium in the interaction between periodontal cells and bacteria found in the mouth, in particular in dental deposit. The pathogenic processes typical of gingivitis and periodontitis are caused by the immunopathological reaction, which, in turn, is initiated by bacteria present in dental deposit. This reaction leads to a progressing wavelike chronic inflammation, which is accompanied by the development of destructive processes. 

The regulation of the inflammatory response is known to play an important role in the pathogenesis of gingivitis and perodontitis [3]. A study of transcriptome changes in the gingival biopsies during the development and treatment of experimental gingivitis showed that, during the development of the disease, there are changes in the expression level of tens of immune response genes, including interleukins * IL1A, IL1B, IL8* and a number of other genes[4]. In this instance, the transcription level of one gene increases, whilst the level of other genes decreases.

The role of an individual’s genetic constitution in their predisposition to inflammatory diseases of the periodontal tissue and to the intensity of their progression has been inadequately studied thus far. Association studies reveal that carriers of certain cytokine gene alleles are more prone to develop gingivitis and/or periodontitis [5—9]. However, these data are rather contradictory [6, 10, 11].

 The purpose of the present work is to study the association between the severity of the periodontal disease and the alleles affecting the transcription level of eight human cytokine genes, the level of which determines the severity of the inflammation and destructive processes in periodontal tissues. The results could allow to identify potential risk groups of inflammatory periodontal diseases and can later be used in preventive, personalized medicine. 

##  EXPERIMENTAL 

 A group of 296 male soldiers from 20 to 52 years of age (the average age is 27.0 ± 6.3 years) was examined. The examination was performed during a routine medical examination; the procedure of informed consent was followed; the data were collected on the nationality and place of birth of the volunteers and two generations of their ancestors. The examined group included mainly Russian males (proportion of children from mixed marriages (the percentage of descendants from mixed marriages between Russians, Ukrainians, and Belarusians was 6.5%)). All patients underwent a standard external dental checkup and instrumental examination of the oral cavity. The description of the dental status contained the assessment of the intensity of inflammation and destructive processes and the index of oral hygiene. 

**Table 1:** SNPs of cytokine genes checked in this work

Gene	Chromosome	SNP	dbSNP ID	Function	Ref.
*IFNG*	12q14	+874 Т>A	rs2430561	Increasing the expression of HLA II by antigen-representing cells, increasing the level of intercellular adhesion molecules, increasing the level of proliferation of T-cells and production of Th1-cytokines	[14]
*IL1A*	2q14	-889 C>T	rs1800587	Anti-inflammation cytokine. Activation of osteoclasts, T-cells, and matrix metal proteases	[15, 16]
*IL1B*	2q14	-511 G>A	rs16944		
*IL4*	5q31.1	-590 C>T	rs2243250	Decreasing the level of anti-inflammation cytokine. Involvement in differentiation of B-cells and production of antibodies	[17]
*IL6*	7p21	-174 G>C	rs1800795	Activation of osteoclasts. Involvement in differentiation of B-cells and production of antibodies	[18]
*IL10*	1q31-q32	-592 C>A	rs1800872	Inhibiting replication of T-cells and inhibiting synthesis of anti-inflammation cytokine. Involvement in differentiation of B-cells and production of antibodies	[18]
*IL18*	11q22.2-q22.3	-607 G/T	rs1946518	Anti-inflammation cytokine. Increasing the level of producing IFN-γ by Т-cells.	[19-21]
*TNF *	6p21.3	-308 G>A	rs1800629	Activation of osteoclasts and matrix metal proteases. Increasing the expression of HLA II by antigen-representing cells. Increasing the level of intercellular adhesion molecules	[19-21]

For the assessment of the severity of gingivitis, the PMA (papillary-marginal-alveolar) index was used [12]. In order to determine this index, the condition of gum tissue for each tooth was assessed in points after it was stained with the purpose of revealing the inflamed areas; the value averaged over all teeth was calculated. PMA = 0 corresponds to the absence of gingivitis, PMA values of up to 30% inclusive correspond to mild gingivitis, the range from 31% to 60% corresponds to gingivitis of moderate severity, whereas PMA values of more than 60% correspond to severe gingivitis.

 The examination of the probing pocket depth (PPD) was performed using a special periodontal probe. The highest value of the PPD (mm) was registered, and all values were then summarized and categorized by the number of teeth examined. 

The hygiene condition of the oral cavity (the amount of dental calculus and dental deposit) was assessed in accordance with the OHI-S (oral hygiene indices – simplified) index [13].

Samples of venous blood were collected into evacuated tubes containing EDTA. DNA was extracted from the blood samples using the standard phenol-chloroform method. The alleles of the genes analyzed were genotyped twice using an “Immunogenetics” kit (DNA-technology, Moscow). PCR amplification and genotyping were carried out in 384 well plates using a DT-384 amplifier (DNA-technology, Moscow). Eight cytokine genes were investigated: γ-interferon (IFN-γ); α- and β-subunits of interleukin 1 (IL-1α and IL-1β); IL-4, IL-6, IL-10, IL-18 interleukins; and tumor necrosis factor α (TNFα) ( *Table 1* ). Polymorphic SNPs predominantly from the promoter regions, affecting the expression of the indicated genes, were selected for analysis. The polymorphic SNPs are listed in *Table 2* .

**Table 2:** Mean values of the dental indices in carriers of some genotypes over eight cytokine genes

Genotype		N	Dental indices (±SE)*			Minor allele effect**
			PMA	PPD	OHI-S	
*IFNG *(+874)	A/A	67	0.25±0.02	1.61±0.10	1.85±0.09	*p *> 0.2 for all indices
	T/A	127	0.22±0.01	1.42±0.06	1.74±0.07	
	T/T	87	0.24±0.04	1.48±0.07	1.78±0.07	
*IL1A *(-889)	C/C	140	0.22±0.01	1.40±0.06	1.72±0.06	PMA↓ Recessive*р *= 0.026
	C/T	118	0.26±0.02	1.59±0.07	1.84±0.07	
	T/T	27	0.20±0.02	1.45±0.11	1.79±0.12	
*IL1B *(-511)	A/A	29	0.26±0.03	1.41±0.11	1.77±0.12	PMA↑ Recessive*р *= 0.157
	G/A	122	0.23±0.02	1.44±0.07	1.80±0.07	
	G/G	133	0.22±0.01	1.49±0.06	1.76±0.06	
*IL4 *(-590)	C/C	171	0.24±0.01	1.49±0.06	1.79±0.05	*р *> 0.5 for all indexes
	C/T	98	0.23±0.02	1.45±0.07	1.78±0.07	
	T/T	14	0.19±0.04	1.51±0.22	1.57±0.20	
*IL6 *(-174)	C/C	61	0.25±0.02	1.53±0.10	1.70±0.07	PMA↑ Dominant*p *= 0.003
	G/C	139	0.24±0.01	1.48±0.06	1.83±0.07	
	G/G	85	0.21±0.02	1.46±0.08	1.74±0.08	
*IL10 *(-592)	A/A	23	0.24±0.03	1.57±0.15	1.66±0.16	OHI-S↓ Dominant*р *= 0.043
	C/A	110	0.22±0.01	1.46±0.07	1.72±0.06	
	C/C	152	0.24±0.01	1.49±0.06	1.84±0.06	
*IL18 *(-607)	C/C	85	0.25±0.02	1.50±0.08	1.69±0.07	OHI-S↑ Recessive*р*= 0.022; PMA↑ recessive*р *= 0.188
	A/C	140	0.21±0.01	1.44±0.06	1.75±0.06	
	A/A	57	0.27±0.02	1.57±0.09	1.96±0.09	
*TNF *(-308)	A/A	4	0.17±0.04	1.45±0.22	1.83±0.25	*р *> 0.2 for all indexes
	G/A	68	0.25±0.02	1.50±0.08	1.84±0.09	
	G/G	212	0.23±0.03	1.48±0.05	1.76±0.05	

*The association level is significant.

**The arrow indicates an increased or decreased value of the character for the minor allele carriers (dominant effect) and for homozygous minor allele carriers (recessive effect). The minimum levels of significance are calculated in accordance with the permutation test.

A statistical analysis was carried out by the standard methods using the WinSTAT 2003.1 software integrated into the Excel software. For the intergroup comparison of the dental indices, the nonparametric Mann-Whitney criterion was used. We used WinPepi freeware, *http://www.brixtonhealth.com/pepi4windows.html * [22],to estimate theodds ratio (OR) and the significance of the differences between the frequencies in accordance with the Fisher’s exact test. Corrections for multiple comparisons were introduced by the permutation test (100,000 stimulations) performed using the Mathematica 5.1 software.

##  RESULTS AND DISCUSSION 

 In only 1% (three patients) of the group examined (296 males), no sign of inflammation of gum tissue (PMA = 0) was observed Mild gingivitis was found in 67.6% of males (205 patients), and 31.4% of males were suffering from gingivitis of moderate severity (93 patients) and severe gingivitis (12 patients) (PMA > 30%). The probing pocket depth varied from 0.6 to 4.5 mm. The mean value across the group examined was 1.50 ± 0.73 mm (±S.D.). The values of the OHI-S indices was in a range from 0 to 3.8 points, the mean value was 1.79 ± 0.73; it corresponds to an unsatisfactory hygiene level. The genotypes of all males examined were determined for each from eight loci. Due to technical difficulties, the genotypes of some SNPs were not determined for each individual; hence, the total number of genotypes for each gene is different. 

**Fig. 1:**
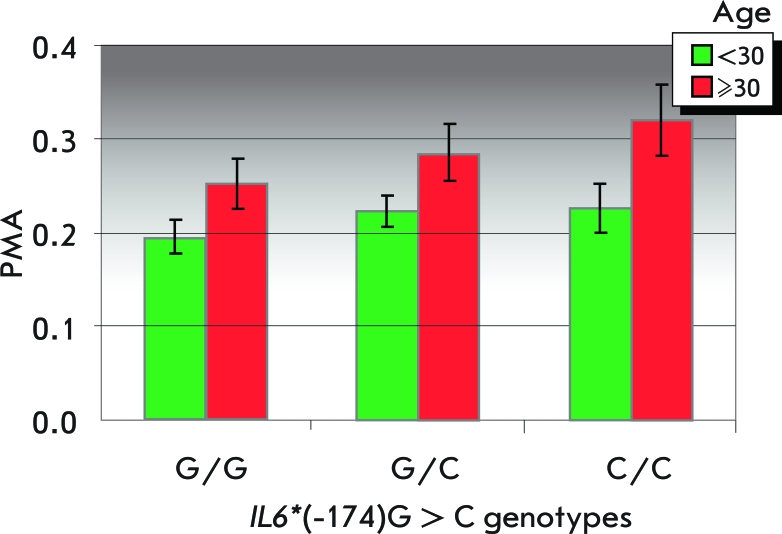
Mean values (±SE) of the PMA index in carriers of different *IL6 * (-174) genotypes from two groups: less than 30 years old, 215 individuals; and more than 30 years old, 70 individuals.

 The dental indices can be considered as quantitative characters; hence, in order to assess how genes influence the value of the characters, the mean values of the indices for the investigated genes were calculated in carriers of each genotype. The significant differences between the mean values of the indices for carriers of different genotypes indicate the possible influence of a gene on a given character. In this case, the differences between the carriers of the considered allele and the carriers not having this allele (homozygous alternative allele carriers) indicate the dominant effect of the considered allele and the recessive effect of the alternative allele. An increase in character, depending on the number of copies of the allele in the genotype (0, 1 or 2), corresponds to the additive model (the significance of the regression of the character value per number of allele copies is determined). 

The mean values of dental indices were calculated in carriers of different genotypes over eight cytokine genes ( *Table 2* ). The significance levels of the effects of minor alleles calculated by the permutation test are contained in the right column of *Table 2* . The effects of minor alleles were estimated simultaneously in 100,000 permutations in accordance with the dominant, recessive, and additive models. The results of the significance were used in order to further verify the significance of associations. The additive model gave no added significance compared with the analysis results on the recessive/dominant effects; thus, the data for the additive model are not listed.

A significant increase in the mean PMA index value was revealed in carriers of the IL6* (-174)С allele ( *Table 2* ). Since the PMA index value correlates with the age of patients ( *r* = 0.16 at *p* = 0.004), the influence of the gene was checked in two age groups: 215 patients were up to 30 years old, and 70 patients were 30 years old and older). A significance association of the IL6* (-174)С allele with an increased PMA index value according to the dominant model is revealed in each age cohort ( *Fig. 1* ). When splitting each age cohort into groups with high PMA (>30% corresponds to moderate and severe gingivitis) and low (<30% corresponds to normal and mild gingivitis), the carriers of the IL6 * (-174) C allele showed a greater risk of being in the group of moderate and severe gingivitis. The corresponding odds ratios were *OR* = 2.22 at *p* = 0.031 in the group of patients younger than 30 and *OR* = 3.78 at *p* = 0.052 in the group of patients 30 years old and older. The uniformity of the effects in both age groups (the differences in the *OR * are negligible, *p* = 0.496 according to the χ ^2^ criterion) allows to estimate the odds ratio in the pooled sample: *OR* = 2.56 at *p* = 0.002 (95%CI = 1.32—5.21).

Analogous calculations carried out for the *IL1A* *(-889) SNP revealed that the carriers of the major C allele are in greater risk of suffering from moderate and severe gingivitis (PMA > 30%) in comparison to the carriers of the T/T genotype: *OR* = 3.86 at *p* = 0.026 (95%CI = 1.14—13.10).

In addition to the assessment of the individual gene effects, their pairwise action was considered. The pairwise action of the minor alleles in the IL6* (-174) SNP of the *IL6 * gene and the IL1B* (-511) SNP of the *IL1B * gene was found, while no significant effects of these genes were revealed in the previous stage of analysis. During consideration of the pairwise action of these two genes, their influence on the mean PMA value and on the association with moderate and severe gingivitis was analyzed as in the previous stage.

**Fig. 2:**
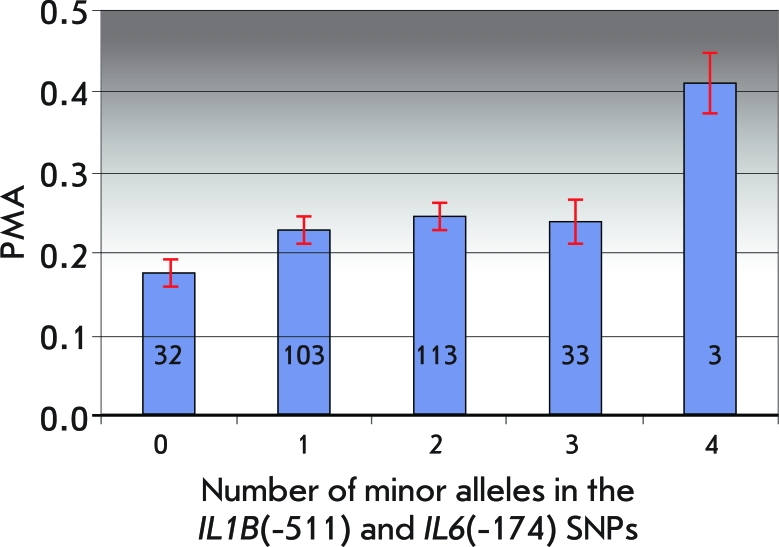
Mean values (±SE) of the PMA index vs. the number of minor alleles in the *IL1B * (-511) and *IL6 * (-174) SNPs. The number of individuals carrying each genotype is shown in histogram bars.

The lowest mean PMA value was typical of double homozygotes for both SNPs. Upon increase in the number of minor alleles in the IL1B* (-511) and  IL6* (-174) SNPs, the mean PMA index value increases ( *Fig. 2* ). The difference in the PMA index values (by a factor of 2.3) found for two extreme cases (double homozygotes for the minor alleles and homozygotes for the major alleles) are significant according to the Mann-Whitney test ( *p* = 0.0067).

**Fig. 3:**
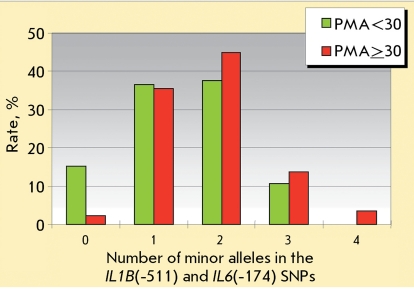
Distribution of the total number of minor alleles copies found in the SNP *IL1B * (-511) and *IL6 * (-174) SNPs vs. severity of gingivitis. The group of individuals with PMA index ≥ 30 (moderate and severe gingivitis) includes 87 individuals. The group of individuals with PMA index < 30 (no gingivitis and mild gingivitis) includes 197 individuals.

The distributions of the total number of minor allele copies in the IL1B* (-511) and  IL6* (-174) SNPs in the patients suffering from severe and moderate gingivitis (PMA ≥ 30%), from mild gingivitis (PMA < 30%), and the healthy patients are shown in Fig. 3. It can clearly be seen that the carriers of at least one minor allele of the IL1B* (-511) and  IL6* (-174) genes are more likely to be found amongst patients with severe gingivitis. The risk of development of moderate and severe gingivitis (PMA ≥ 30%) is higher in the homozygous carriers of the minor IL1B* (-511)A and  IL6* (-174)С alleles than in the homozygous carriers of the major IL1B* (-511)G and  IL6* (-174)G alleles and is characterized by an odds ratio *OR * = 7.63 at *p* = 0.0009 (95%CI = 1.80—32.45). The percentage of homozygous carriers of minor alleles (individuals carrying the combination of the A/A genotype of the IL1B* (-511) SNP and C/C genotype of IL6* (-174) SNP associated with severe gingivitis) was 1.1%; the percentage of individuals carrying the “protective” combinations of the G/G and G/G genotypes of the same SNPs was 11.3% (32 patients). This effect has to be confirmed in larger samples, but an increase in the effect occurring upon an increase in the number of minor alleles argues for the nonarbitrariness of the association revealed ( *Fig. 2* ).

The prevention and treatment of periodontal diseases is a very pertinent issue within dentistry. Periodontal diseases are found even in infancy, and apparent clinical manifestations of this pathology are observed in more than half of people in their 30s. However, the problems of prevention and effective treatment of periodontal diseases remains unsolved [23]. The manifestations of the clinical signs of this disease depend on age, pernicious habits, general health status, and overall hygiene. In this context, it is reasonable to consider the phenomenon of the association between the OHI-S index and SNPs in the interleukin genes. The OHI-S index is increased in homozygous carriers of the *IL10* *(-592)C allele (its value is 1.84 ± 0.06 against 1.71 ± 0.06 in carriers of the allele A; however the difference is insignificant, since *p* = 0.092 according to the Mann-Whitney test). Significant differences are observed in individuals carrying different genotypes of the IL18* (-607) SNP, G > A. The value of the OHI-S index in carriers of the homozygous A/A genotype is 1.96 ± 0.09, while in carriers of the G allele, its value is 1.73 ± 0.05 (the difference is significant, *p* = 0.018 according to the Mann-Whitney test). It may be assumed that since hygiene hardly depends on the genotype, in homozygous carriers of the IL18* (-607) А allele, the formation of plaques dental deposit is more intensive and pushes up the value of the OHI-S index.

 No association of the PPD with the alleles of cytokine genes was found. 

In this work, we established an association between the IL1A* ( *-* 889)Сand  IL6* ( *-* 174)С alleles and the severity of gingivitis and the increase in the effect of the IL6* ( *-* 174)С allele in the presence of the IL1B* ( *-* 511)A allele. The 511 A/G polymorphism influences the expression of the *IL1B * gene under certain conditions and is associated with inflammatory and cancer diseases [11, 24]. Interleukin 1 is an anti-inflammatory cytokine secreted by monocytes, macrophages, and dendritic cells. The *IL1* geneis one of the first genes for which an association of single-nucleotide polymorphisms with inflammatory diseases of periodontal tissue was revealed in [5]. In the development of a periodontal disease, the role of interleukin 1 involves the induction of inflammation mediators. It has been demonstrated that in immortalized human gingival fibroblasts, the transcription level of the inflammatory genes of cytokines, chemokines, metalloproteases, cell adhesion molecules, and the transcription factor NF-kβ, which controls the expression of immune and anti-apoptotic response genes and cell cycle genes, increase. The activation of NF-kβ blocks apoptosis, thereby causing the stabilization of gingival fibroblasts *in vitro* [25]. An association of the polymorphic markers in the interleukin 1 gene cluster ( *IL1A* , *IL1B* and  *IL1RN* , IL1 receptor antagonist) with periodontitis and gingivitis has been established only in a few works [11].

Interleukin 6 is a multifunctional cytokine that plays an important role in the inflammatory response on infectious agents (especially gram-negative bacteria) [26]. The production of cytokine is decreased in carriers of the minor C allele at the -174 position of the regulatory site in the IL6 gene; this may cause a destruction of the immune protection [27]. While studying the associations of both genes with the inflammation of periodontal tissue, contradictory data were obtained. An analysis of the association between chronic periodontitis and the *IL6* (-174) SNP performed in six Caucasian populations reveal the presence of the association in three of them [11]. The interleukin genes may be involved in the regulation of the inflammatory response and could be responsible for the differences observed in the spectrum of oral pathogens for carriers of different genotypes. The *Aggregatibacter actinomycetemcomitans * and * Porphyromonas gingivalis * pathogenic bacteria were revealed to be more frequently found in sublingual biofilms for the carriers of a certain genotype in the *IL6* (-174) SNP [8]. The contradictions in the results obtained by different authors can be explained by changes in the correlation between the factors depending on age, ethnic homogeneity, and other peculiarities of the groups examined.

The formation of dental calculus and dental deposit is a complex process that depends on the quality of oral hygiene, microbiota, and local immunity. Genetically determined features of the immune response might also influence the formation of dental deposits and, consequently, the OHI-S index. Interleukin 18 is an anti-inflammatory cytokine, and in all probability it is one of the main cytokines involved in the development of pathological processes and destruction of periodontal tissues; it is secreted by macrophages/monocytes and epithelial cells of the oral cavity, stimulates cellular immunity and induces the production of IFN-γ. The level of interleukin 18 is increased in cases of various chronic inflammatory diseases. The content of interleukin 18 in gingival fluid increases proportionally to the severity of a periodontal inflammation and decreases to its initial level after the disease is cured [28, 29]. The -607 C/A SNP in the regulatory site of the *IL18 * gene is localized in the CREB (cAMP response-element binding protein) site [30]. A significant increase in both the spontaneous and lipopolysaccharide-induced productions of interleukin 18 is observed in the cell culture of donors carrying the *A* allele [31]. The association of the OHI-S index with the interleukin gene alleles had not been described earlier. The associations revealed are significant for the ethnically homogeneous sample (Russians) examined. To date, the severity of the inflammation and destructive processes in periodontal diseases is known to be genetically dependent. These diseases should be considered as multifactor diseases [11]. However, the contribution of certain genes to the predisposition to and tolerance of inflammatory diseases of the periodontal tissue requires a more detailed study, especially with different genetic backgrounds, which could be ethnically dependent. Therefore, the practical significance of the association revealed here makes testing these results in independent samples of other ethnic groups especially interesting.
